# Racial differences in lifestyle, demographic, and health factors associated with quality of life (QoL) in midlife women

**DOI:** 10.1186/s40695-020-00060-1

**Published:** 2021-01-06

**Authors:** Brandi Patrice Smith, Esmeralda Cardoso-Mendoza, Jodi A. Flaws, Zeynep Madak-Erdogan, Rebecca L. Smith

**Affiliations:** 1 Illinois Informatics, University of Illinois, Urbana-Champaign, Champaign, IL USA; 2 Department of Molecular and Cellular Biology, University of Illinois, Urbana-Champaign, IL USA; 3 Department of Comparative Biosciences, University of Illinois, Urbana-Champaign, Urbana, IL USA; 4 Carl R. Woese Institute for Genomic Biology, University of Illinois, Urbana-Champaign, Urbana, IL USA; 5 Food Science and Human Nutrition Department, University of Illinois, Urbana-Champaign, Urbana, IL USA; 6 Carle Illinois College of Medicine, University of Illinois, Urbana-Champaign, Urbana, IL USA; 7 Cancer Center at Illinois, University of Illinois, Urbana-Champaign, Urbana, IL USA; 8 Beckman Institute for Advanced Science and Technology, University of Illinois, Urbana-Champaign, Urbana, IL USA; 9 National Center for Supercomputing Applications, University of Illinois, Urbana-Champaign, Urbana, IL USA; 10 Department of Pathobiology, College of Veterinary Medicine, University of Illinois, Urbana-Champaign, 2001 S. Lincoln Ave, Urbana, IL USA

**Keywords:** Quality of life, Midlife, Menopause, Disparities, Race

## Abstract

Previously, quality of life (Qol) has been defined as an individual’s evaluation of a satisfactory life as a whole (i.e. physically, mentally, psychologically, and socially). Only a few studies have examined the racial differences between QoL and risk factors associated with health, demographics, and lifestyle in midlife women. Thus, the purpose of our study was to determine racial differences in QoL in menopausal women due to lifestyle, demographic, and health related risk factors. A stratified ordinal logistic regression model was applied to self-reported questionnaire data from the Midlife Women’s Health Study (MWHS) to determine risk factors associated with QoL differences between White and Black women during the menopausal transition. In multivariable models, our results showed Black women who had 3 or 4 comorbidities were about 4 times as likely to have higher QoL compared to women who had 0 to 2 comorbidities (95% CI: 1.65,10.78). However, the number of comorbidities was not significantly associated with QoL in White women in univariate or multiple regression. Further, body mass index and income were not significant factors in QoL in Black women but were in White women. Overall, our results illustrate that differences in health, demographic, and lifestyle factors are associated with QoL during menopause. Also, we suggest that future studies evaluate stratified models between racial groups to determine race-specific risk factors related to quality of life.

**Supplementary Information:**

The online version contains supplementary material available at 10.1186/s40695-020-00060-1.

## Background

Previously, quality of life (Qol) has been defined as an individual’s evaluation of a satisfactory life as a whole (i.e. physically, mentally, psychologically, and socially) [1, 2]. The midlife period, in which most women begin to experience the menopausal transition, has been shown to have negative impacts on QoL [3–5]. The decline in QoL could be attributed to stark decreases in estrogen production and the onset of one or many metabolic diseases during menopause [6, 7]. During the menopausal transition, women often experience irregular periods, hot flashes, trouble sleeping, and pain during sex [3–6].

When studying midlife women, it is important to consider the racial differences in risk factors and QoL, as these relationships could help explain racial differences in perceptions of health and determine systemic factors which play a role in those differences [8, 9]. The number of publications on QoL in midlife women regarding race is limited. A recent study has found that middle-aged Black women are biologically older (by about 7.5 years) than White women of the same chronological age due to Black women’s greater exposure to stressors which can negatively impact QoL [10]. Furthermore, in Black women, body mass index (BMI) has been shown to have a negative linear relationship with health-related QoL, compared to the inverse-U shape for White women [11]. Black women have consistently higher BMI levels through all levels of education, whereas White women appear to have decreased BMI with increasing education levels. This can be of concern because normal range BMI has been associated with a lower probability of vasomotor symptoms in menopausal women as well as higher scores on health-related QoL [12].

Well-known disparity factors, such as socioeconomic status (SES), may also have negative impacts on QoL. Regardless of race, low SES poses an increased risk of poor health and higher odds of lifetime morbidity when compared to high SES among middle-aged women. The odds of poor outcomes greatly increase when a woman is both Black and of low SES [13]. Even when Black and White women are of the same SES, Black women report a greater number of lifetime morbidities when compared to their White counterparts [13]. The health and socio-demographic factors disparately affecting Blacks can cause a great variation in perceived life satisfaction. It is important to acknowledge these disproportionalities to be able to identify risk factors for low QoL. Thus, the objective of this study is to determine which health, demographic, and lifestyle factors differ in QoL between Black and White women.

## Methods

### Study sample

The sample population included women from the Midlife Women’s Health Study (MWHS), which has been described in detail elsewhere [14]. The MWHS is a longitudinal study that was conducted between 2006 and 2015 to understand the relationship between risk factors and hot flashes in midlife women. Thus, women who were identified as post-menopausal were excluded from the study. The MWHS study population consisted of women between the ages of 45–54 from Baltimore, Maryland, and surrounding areas. Eligible women engaged in a baseline clinic visit where they completed a survey questionnaire and several biological measures were taken, including height and weight. Self-reported responses from the baseline survey questionnaire were utilized for analysis in the present study. The MWHS was previously approved by the University of Illinois and Johns Hopkins University Institutional Review Board. All participants provided informed consent for the study. Women were divided into subgroups based on race to understand racial differences in quality of life. Latinas, Native Americans, and Asian Pacific Islanders were excluded from the analysis because of small sample sizes (*n* < 30). Only self-identified Black and White participants were included in the final analysis.

### Study variables

Several demographic, lifestyle and health factors from the MWHS questionnaire were included in the study. The QoL indicator was derived from Cantril’s Ladder of Life, a self-anchoring scale that measures a person’s attitude toward their health [15]. The question posed in the survey was “Here is a ladder representing the ‘Ladder of Life.’ The top of the ladder represents the best possible life for you. The bottom of the ladder represents the worst possible life for you. On which step of the ladder do you feel you personally stand at the present time?” The scale ranges from 1 to 10, with 1 representing the “worst possible life” and 10 representing the “best possible life”. QoL was stratified by low (1–4), middle (5–7), and high (8–10) levels to control for variation in the frequency of choice in scale values.

Demographic factors of interest included were age, annual family income, marital status, education level, and employment status. Each variable was dichotomized to a binomial factor except annual income which was partitioned to a tercile variable: Low (<=$34,999), Middle ($35,000–$74,999), and High (> = $75,000). Lifestyle factors or modifiable factors that can greatly influence health included BMI, smoking status, and drinking status [16]. BMI was calculated from measured height and weight then characterized as normal/underweight(< 25 kg/m2), overweight (between 25 and 29 kg/m2), or obese (> 30 kg/m2) [14]. Drinking status was determined by responses to the following question, “In the last 12 months have you had at least 12 drinks of any kind of alcoholic beverage?”, bivariate responses of yes or no were recorded.

Health factors included in the study were depression, number of comorbidities, menopausal status, hot flash experience, hormone replacement therapy use, pregnancy status, sexual activity, and sleep disturbances. Depression was measured using the Center for Epidemiologic Studies Depression Scale (CES-D) survey [17]. CES-D score was used to identify depressive symptoms in participants. CES-D scores of < 16 were considered not depressed and > =16 were considered depressed. Women were asked if they had been diagnosed with any of the following potential morbidities: diabetes, heart disease, stroke, hypertension, high cholesterol, anemia, breast cancer, ovarian cancer, uterine cancer, other cancer, epilepsy, lupus, thyroid disorder, depression, cataracts, stomach ulcer, yellow jaundice, cirrhosis of the liver, hepatitis, arthritis, allergies, asthma, hay fever, eczema, rosacea, psoriasis, fibroids or other skin disorder. The number of comorbidities, self-reported from this list, was categorized as 0–2, 3–4, or > 5 comorbidities. Menopausal status was defined by the number of periods a woman experienced at the time of reporting [14]. Premenopausal women were defined as women who had their last menstrual period within the past 3 months and reported 11 or more periods within the last year, perimenopausal women were defined as women having their last menstrual period within the past year, but not within 3 months or who had their last period within the past 3 months and 10 or fewer periods within the past year, and lastly postmenopausal women were defined as having no periods within the past year (*N* = 1), thus this observation was omitted. Menopausal status was dichotomized by peri- and pre-menopausal. Hot flashes, hormone replacement therapy use, history of pregnancy, and sexual activity were categorized into “yes” or “no” based on whether a woman experienced the factor or not. Sleep disturbances were categorized by frequency of disturbances: “never-4 times per month”, “2-4 times per week”, and “>5 times per week”.

### Statistical analysis

Ordinal logistic regression was applied to analyze the relationship between QoL and possible risk factors (demographic, lifestyle, and health factors). Assumptions for no multicollinearity and proportional odds were tested using the Brant Test function from the *brant* package in R [18]. Univariate models were conducted to determine the individual effects of each factor on QoL. An alpha value of 0.05 was considered statistically significant. Due to the high levels of missingness of responses and lack of significance in univariate analysis, drinking and hormone replacement therapy were omitted from multiple regression for both populations.

Multivariate regression models were fitted by backward, stepwise regression based on the BIC [19]. Odds ratios greater than 1 implied increased likelihood of high quality of life. Regression analysis for univariate and multivariable models was stratified by race to compare relationships of QoL within each race. Black-White comparisons are important because the widest gap in health disparities occurs between these two populations [20]. Analyses were conducted using R statistical software version 3.5.3 [21] and the *polr* function in the MASS package [22].

To determine the reliability of our fitted models we performed bootstrapping by the AIC in R [23]. During the bootstrapping process, we simulated data sets for each stratified population by sampling with replacement at 100 iterations. Next we re-fitted each simulated set to determine the number of times our covariates of interest were selected for the model [24]. In multivariable models of White women, CES-D, income, and BMI were selected 100, 93 and 92% of the time in refitted models, respectively. Furthermore, the number of comorbidities, smoking status, CES-D, and marital status were selected 90, 89, 88, and 79% of the time in refitted models of Black women, respectively (Supplemental Table 1).

Post-hoc analysis of the comorbidity-QoL relationship was examined using network analysis and determining the frequency of morbidity occurrence. Networks were constructed in R using the package igraph [25]. Nodes were represented by individual morbidities and edges were represented by co-occurrences of morbidities within each population. Larger nodes represented morbidities that occurred more frequently.

## Results

### Unadjusted associations of QoL and health, demographic and lifestyle factors

Distributions of risk factors and unadjusted association with QoL are shown in Tables 1 and 2. Income, marital status, and CES-D were significantly associated with QoL in both Black and White women. However, differences between the two groups existed. For White women only, education (*p* = 0.003), BMI (*p* = 0.003), smoking status (*p* = 0.039), ever pregnant (*p* = 0.049), sleep disturbances (*p* < 0.001), and being sexually active (*p* < 0.001) were significantly associated with QoL.

**Table 1:** Unadjusted associations of QoL in White women

	White			
Risk Factor	lowQOL, *N* = 46^1^	midQOL, *N* = 142^1^	highQOL, *N* = 239^1^	*p*-value^2^
**Age Group**				
45–49	27 (59%)	93 (65%)	154 (64%)	0.7
50–54	19 (41%)	49 (35%)	85 (36%)	
**Education Level**				
Graduated college	26 (57%)	101 (71%)	190 (79%)	0.003
Did not graduate college	20 (43%)	41 (29%)	49 (21%)	
**Income Level**				
High Income	16 (35%)	97 (68%)	190 (79%)	< 0.001
Low Income	9 (20%)	8 (5.6%)	10 (4.2%)	
Middle Income	21 (46%)	37 (26%)	39 (16%)	
**BMI Status**				
obese BMI of 30 or greater	16 (35%)	37 (26%)	40 (17%)	0.003
overweight BMI of 25–29.9	8 (17%)	44 (31%)	56 (23%)	
normal BMI of 18.5–24.9	22 (48%)	61 (43%)	143 (60%)	
**Marital Status**				
Married	25 (54%)	101 (71%)	188 (79%)	0.002
Single	11 (24%)	20 (14%)	16 (6.7%)	
Widowed/Divorced/Separated	10 (22%)	21 (15%)	35 (15%)	
**Smoking Status**				
Current	9 (20%)	14 (9.9%)	17 (7.1%)	0.039
Former/Never	37 (80%)	128 (90%)	222 (93%)	
**Drinker**	37 (80%)	112 (79%)	210 (88%)	0.053
**Employment Status**				
Employed	32 (70%)	117 (82%)	198 (83%)	0.10
Unemployed	14 (30%)	25 (18%)	41 (17%)	
**Menopausal Status**				
Peri-menopause	21 (46%)	64 (45%)	85 (36%)	0.13
Pre-menopause	25 (54%)	78 (55%)	154 (64%)	
**Experienced Hot flashes**				
Yes	23 (50%)	68 (48%)	93 (39%)	0.14
No/Don’t Know	23 (50%)	74 (52%)	146 (61%)	
**No. of Comorbidities**				
0–2	16 (35%)	66 (46%)	122 (51%)	0.061
3–4	15 (33%)	44 (31%)	80 (33%)	
5 or more	15 (33%)	32 (23%)	37 (15%)	
**CES-D > =16**	31 (67%)	47 (33%)	18 (7.5%)	< 0.001
**Hormone Replacement Therapy Use**	2 (4.3%)	1 (0.7%)	6 (2.5%)	0.2
**Ever Pregnant**	35 (76%)	128 (90%)	208 (87%)	0.049
**Sleep Disturbances**				
> 5 times per month	12 (26%)	14 (9.9%)	27 (11%)	< 0.001
0–4/mon	17 (37%)	92 (65%)	166 (69%)	
2–4 times per month	17 (37%)	36 (25%)	46 (19%)	
**Sexually Active**	32 (70%)	114 (80%)	215 (90%)	< 0.001

^1^Statistics presented: n (%)

^2^Statistical tests performed: chi-square test of independence; Fisher’s exact test

**Table 2:** Unadjusted associations of QoL in Black women

	Black			
Risk Factor	lowQOL, *N* = 22^1^	midQOL, *N* = 41^1^	highQOL, *N* = 78^1^	*p*-value^2^
**Age Group**				
45–49	19 (86%)	25 (61%)	52 (67%)	0.11
50–54	3 (14%)	16 (39%)	26 (33%)	
**Education Level**				
Graduated college	7 (32%)	23 (56%)	32 (41%)	0.13
Did not graduate college	15 (68%)	18 (44%)	46 (59%)	
**Income Level**				
High Income	4 (18%)	12 (29%)	39 (50%)	< 0.001
Low Income	11 (50%)	6 (15%)	17 (22%)	
Middle Income	7 (32%)	23 (56%)	22 (28%)	
**BMI Status**				
obese BMI of 30 or greater	14 (64%)	28 (68%)	41 (53%)	0.2
overweight BMI of 25–29.9	5 (23%)	12 (29%)	25 (32%)	
normal BMI of 18.5–24.9	3 (14%)	1 (2.4%)	12 (15%)	
**Marital Status**				
Married	3 (14%)	16 (39%)	46 (59%)	0.001
Single	11 (50%)	18 (44%)	19 (24%)	
Widowed/Divorced/Separated	8 (36%)	7 (17%)	13 (17%)	
**Smoking Status**				
Current	8 (36%)	8 (20%)	10 (13%)	0.053
Former/Never	14 (64%)	33 (80%)	68 (87%)	
**Drinker**	16 (73%)	30 (73%)	51 (65%)	0.6
**Employment Status**				
Employed	14 (64%)	33 (80%)	67 (86%)	0.067
Unemployed	8 (36%)	8 (20%)	11 (14%)	
**Menopausal Status**				
Peri-menopause	7 (32%)	17 (41%)	35 (45%)	0.5
Pre-menopause	15 (68%)	24 (59%)	43 (55%)	
**Experienced Hot flashes**				
Yes	13 (59%)	20 (49%)	34 (44%)	0.4
No/Don’t Know	9 (41%)	21 (51%)	44 (56%)	
**No. of Comorbidities**				
0–2	9 (41%)	14 (34%)	24 (31%)	0.2
3–4	3 (14%)	13 (32%)	33 (42%)	
5 or more	10 (45%)	14 (34%)	21 (27%)	
**CES-D > =16**	12 (55%)	11 (27%)	9 (12%)	< 0.001
**Hormone Replacement Therapy Use**	0 (0%)	1 (2.4%)	1 (1.3%)	> 0.9
**Ever Pregnant**	20 (91%)	38 (93%)	75 (96%)	0.4
**Sleep Disturbances**				
> 5 times per month	6 (27%)	6 (15%)	13 (17%)	0.7
0–4/mon	11 (50%)	25 (61%)	50 (64%)	
2–4 times per month	5 (23%)	10 (24%)	15 (19%)	
**Sexually Active**	14 (64%)	29 (71%)	66 (85%)	0.055

^1^Statistics presented: n (%)

^2^Statistical tests performed: chi-square test of independence; Fisher’s exact test

### Multivariate associations between QoL and health, demographic and lifestyle factors

**Fig. 1:**
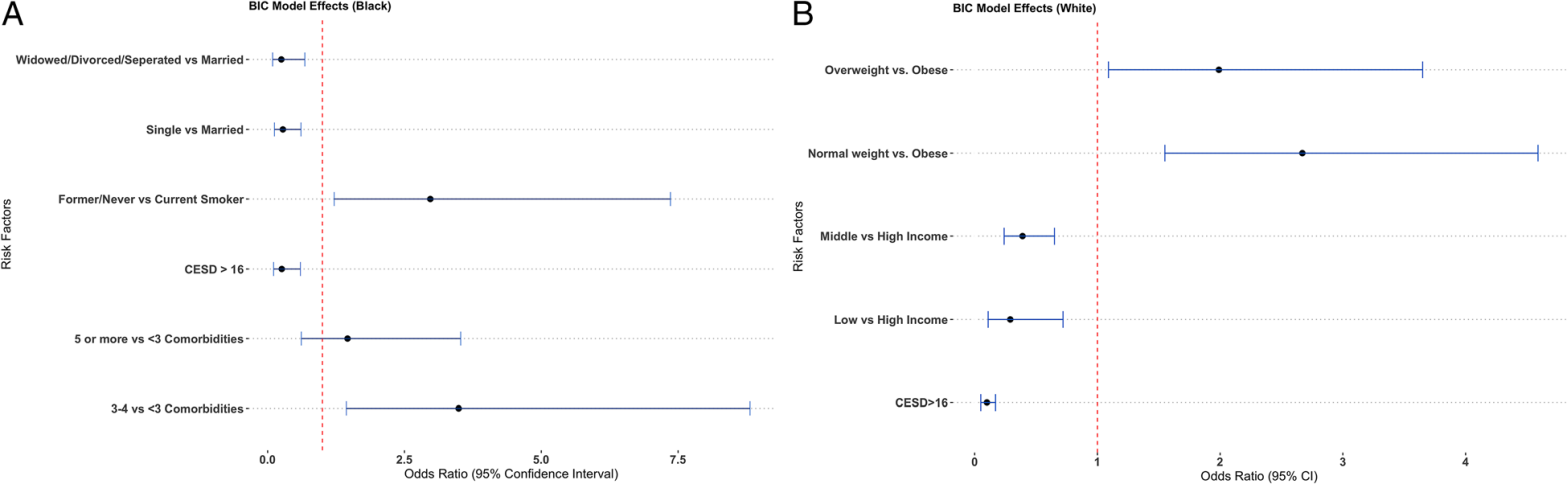
Results of model selection for quality of life in midlife Black (**a**) and White (**b**) women. Points indicate odds ratios, with bars representing the 95% confidence intervals. Dashed line shows odds ratio = 1 (no impact). **a** Multivariate Associations between QOL and Health, Demographic and Lifestyle Factors of Black Women. **b** Multivariate Associations between QOL and Health, Demographic and Lifestyle Factors of White Women

Figure 1a illustrates the odds ratios and 95% confidence intervals of multivariable associations for Black women. Marital status, smoking status, number of comorbidities, and depression factors were chosen for the final model by stepwise regression. Black women who were widowed, divorced or separated were 3.7 times less likely to have high QoL compared to Black married women (95% CI: 1.3, 11.11) and Black single women were also 3.7 times less likely to have high QoL compared to Black married women (95% CI: 1.82, 9.1). Black women who were former smokers or who had never smoked were almost 4 times as likely to have higher QoL compared to Black women who currently smoked (95% CI: 1.5,10.95). Black women who were defined as depressed (CES-D score > 16) were 4.76 times less likely to have high QoL compared to women who were not defined as depressed (95% CI: 2,12.5). Further, Black women who had 3 or 4 comorbidities were 4.12 times as likely to have higher QoL compared to Black women who had 0 to 2 (95% CI: 1.65,10.78). Similarly, Black women who had 5 or more comorbidities were almost 2 times as likely to have higher QoL compared to Black women who had 0 to 2 comorbidities, but this was not significant (95% CI: 0.65, 3.98).

Figure 1b illustrates the odds ratios and 95% confidence intervals of multivariable associations for White women. BMI, income and depression factors were chosen for the final model through backward, stepwise regression. White women with low incomes were 3.44 times less likely to have high QoL compared to women with high income (95% CI: 1.39,9.09) and women with middle incomes were 2.56 times less likely to have high QoL compared to women with high income (95% CI: 1.54, 4.17). Women who were defined as “depressed” (CES-D score > 16) were 10 times less likely to have high QoL compared to women with a CES-D score < 16 (95% CI: 5.88, 20).

### Comorbidity network and frequency of occurrences

**Fig. 2:**
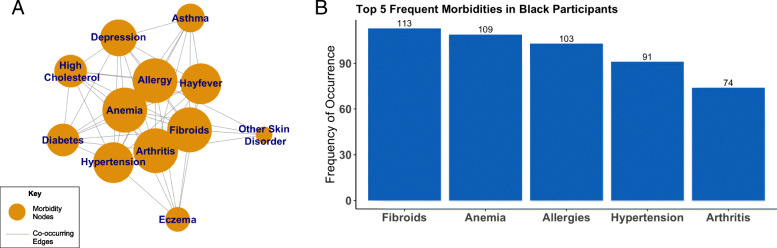
Comorbidity network for a group of Black women in midlife. Node size represents relative frequency of occurrence (**a**). A bar graph illustrating the 5 most frequent morbidities occurring in Black women. The number of women reporting each morbidity is reported above each bar (**b**). **a**. Comorbidity Network for Black Women. **b** Top 5 Frequent Morbidities in Black Women

**Fig. 3:**
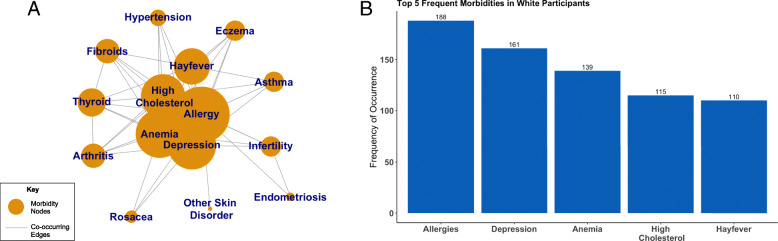
Comorbidity network for a group of White women in midlife. Node size represents relative frequency of occurrence (**a**). A bar graph illustrating the 5 most frequent morbidities occurring in White women. The number of women reporting each morbidity is reported above each bar (**b**). Figure 3**a** title: Comorbidity Network for White Women. **b** Top 5 Frequent Morbidities in White Women

In the comorbidity network for Black women, anemia, arthritis, allergies, hypertension, hay fever, and fibroids cluster together and share high degree values, which indicates they have high co-occurrence with other morbidities (Fig. 2a). The corresponding frequency graph illustrates the five most frequent morbidities in Black women (Fig. 2b) with fibroids being the most frequently reported morbidity (*n* = 113). In the comorbidity network for White women, allergies, high cholesterol, hay fever, anemia, and depression cluster together and have higher degree sizes indicating high co-occurrence with other morbidities (Fig. 3a). White women most commonly reported allergies (*n* = 188) (Fig. 3b). Black women overall had higher numbers of self-reported morbidities than White women.

## Discussion

Our study showed that there are racial differences in the association between quality of life (QoL) during the menopausal transition and risk factors related to lifestyle, demographics, and health. In our final multivariable model, marital status, smoking status, self-perceived depression, and the number of comorbidities significantly varied across QoL in Black women while income and self-perceived depression significantly varied across QoL in White women. We showed these associations by application of ordinal logistic regression separately for White and Black women, identified significant factors for each population, and compared these factors within each population.

Similar results were observed in the Study of Women’s Health Across the Nation (SWAN), which also used stratified models by race/ethnicity to study risk factors in midlife. Being divorced widowed or separated, experiencing very hard financial strain, smoking, and reporting depression were associated with low QoL for African American women in midlife [26]. The SWAN study also reported less than high school education, perceived stress, and social support were associated with low QoL for African American women, but none of these associations were observed in the current study [26]; education was considered in this model but was not retained in the final model fitting, while perceived stress and social support were not measured in this cohort [14]. Additionally, the SWAN study did report statistically significant associations with menopausal status in African American women. The SWAN study also did not include information on morbidity occurrence; thus, a comparison could not be made for this covariate. Additionally, multivariable models for White women demonstrated similar results to our study, they additionally reported less than high school education, self-reported health, heart pounding, smoking, physical activity, attitudes, social support, and surgical menopause was associated with QoL in White women. Finally, BMI was not chosen for final models in the SWAN study for White women [26].

Our results also indicate that being married is a significant indicator of high QoL in Black women [26]. However, a study by Bryant suggests that income and neighborhood setting may be contributing factors explaining the relationship between marital status and high QoL in Black women [27]. This is partially observed in our study; such that unmarried White women had income distributions more similar to married White women compared to unmarried Black women (data not shown), thus indicating the buffering effects of income on QoL in White women. This further validates the complexity of cultural and social norms and their effect on QoL.

Quite surprisingly, in Black women, a moderate increase in the number of comorbidities was significantly associated with the likelihood of higher QoL. This result contradicts previous studies examining the relationship between comorbidities and QoL [28–31]. Comorbidities present as processes which potentially measure allostatic load or wear and tear on the body [32]**.** Our results showed that Black women reported and had a higher frequency of chronic conditions including fibroids (*n* = 113) and hypertension (*n* = 91) compared to White women who were more likely to report depression (*n* = 161) and high cholesterol (*n* = 115). Fibroids are benign, large pelvic tumors that are more common and severe in Black women and are a major public health issue because of costs associated with hysterectomy used to treat the related symptoms such as chronic pain and bleeding [33]. Although a majority of women, Black (80%) and White (70%) may experience fibroids, only a few will have related symptoms and symptoms regress during menopause [33, 34]. This finding may imply that Black women in the current study may not present symptoms from fibroids, thus the demonstrated effect on QoL. Although, some studies attribute the higher fibroid incidence in Black women to having African ancestry, it may be due to their high burden of exposure to environmental chemicals such as phthalates through personal care products [33, 35–37].

Studies show that Black women have a higher prevalence of hypertension compared to White and Hispanic women, thus validating its frequency in the current study [38]. Our study demonstrates Black women have a higher burden of chronic illnesses. A recent study suggested that having health insurance over time is a stronger protective factor from developing chronic diseases in Black women compared to White women [39]. However, in the current study health insurance information was not collected. Additionally, a recent study examining the relationship between comorbidities and QoL found that the “religion and health coping complex” in Black women may be an underlying mechanism explaining the high QoL and comorbidities relationship found here [40]. This coping mechanism enables positive perceptions of health through faith-based activities, even in the presence of multiple chronic morbidities [40, 41]. In the future, capturing access to healthcare and faith-based religious factors may help to further explain this relationship.

Unexpectedly, in contrast to our hypothesis, BMI and education were not significantly associated with QoL in Black women, although those relationships were observed in White women. Previous studies have shown significant relationships between BMI, education, and QoL in Blacks [11, 12]. However, BMI has shown to be an unreliable parameter for obesity and understanding its relationship on QoL, as the association is nonlinear and varies by age for physical QoL but not for mental QoL [42]. This result may also be affected by the fact that Black women are more likely to underestimate their weight compared to White women, even though they have a higher prevalence of obesity [43]; thus their perception of a healthy BMI may differ from the cutoff used in this study. Further effects of BMI perceptions in Black women may be due to cultural norms, diet, weight gain during and after pregnancy, and self-perceived body image [44].

This study has several limitations. First, the QoL measure is subjective or “self-perceived”, although the Cantril’s Ladder of Life has been validated. In addition, this study uses only cross-sectional data. While data were derived from a longitudinal study, the analysis was only conducted for the first year of data. Thus, we have not analyzed any changes in perspective of health as participants move through the menopausal transition. Subsequent analyses will include later years to understand this relationship. Last, Hispanics, Native Americans, and Asians and Pacific Islanders were excluded from the analysis because of small sample sizes (*n* < 30). Thus, analyses were conducted for Black and White women only. In addition, the smaller number of Black women participating (*n* = 141, compared to *n* = 427 for White women) may have resulted in lower power to detect statistically significant associations in Black women.

A major strength of this study was the stratification by race to identify the direct effects of race-specific factors affecting QoL and the validation of our models via bootstrapping. By providing reliable, race-specific models, we are able to identify race-specific risk factors affecting women’s QoL. Additionally, our comorbidity network analysis revealed underlying trends in the number of comorbidities and QoL in Black women in midlife. Previous association studies whose objective was to understand race and QoL routinely include race as a factor in statistical models, which limits their studies to the identification of race and QoL relationships rather than identifying risk factor differences in QoL between races [8, 45].

## Conclusion

Based on our results, we suggest that future studies evaluate stratified models between racial groups to determine race-specific risk factors related to quality of life.

## Supplementary Information

**Additional file 1.** Percentage of Covariates Selected in Bootstrap Re-fitted Models

## Data Availability

The datasets analyzed during the current study are not publicly due to information that would compromise study participants privacy.

## Abbreviations

QoL: Quality of life
MWHS: Midlife women’s health study
AIC: Akaike information criteria
BIC: Bayesian inference criteria
