# Supplementary material for: Racial differences in lifestyle, demographic, and health factors associated with quality of life (QoL) in midlife women
Source: Womens Midlife Health. 2021 Jan 6;7:2. doi: 10.1186/s40695-020-00060-1 (PMC7788772; doi:10.1186/s40695-020-00060-1)
Supplement: Supplementary file 1 — Additional file 1. Percentage of Covariates Selected in Bootstrap Re-fitted Models [file 40695_2020_60_MOESM1_ESM.pdf]

Percentage of Covariates Selected in Bootstrap Re-fitted Models

White		Black	
**Covariate**	**(%)**	**Covariate**	**(%)**
CESD*	100	Comorbidities*	90
Income*	93	Smoke*	89
BMI*	92	CESD*	88
Sexually Active	56	Married*	79
Periods	52	BMI	62
Married	43	Age	54
Comorbidities	31	Periods	54
Parity	30	Sexually Active	48
Education	27	Hotflashes	47
Smoke	27	Sleep Disturbances	35
Sleep Disturbances	26	Income	33
Hotflashes	23	Employment	26
Age	20	Parity	23
Employment	17	Education	22

*covariates with percentages greater than 75 were considered for final models if interesected with stepwise AIC/BIC model
